# Analysis of strain, sex, and diet-dependent modulation of gut microbiota reveals candidate keystone organisms driving microbial diversity in response to American and ketogenic diets

**DOI:** 10.21203/rs.3.rs-2540322/v1

**Published:** 2023-02-02

**Authors:** Anna C. Salvador, M. Nazmul Huda, Danny Arends, Ahmed M. Elsaadi, Anthony C. Gacasan, Gudrun A. Brockmann, William Valdar, Brian J. Bennett, David W. Threadgill

**Affiliations:** Texas A&M University; USDA; Northumbria University; Texas A&M University; Texas A&M University; Albrecht Daniel Thaer-Institut; University of North Carolina; USDA; Texas A&M University

**Keywords:** microbiome, diet, ketogenic

## Abstract

**Background:**

The gut microbiota is modulated by a combination of diet, host genetics, and sex effects. The magnitude of these effects and interactions among them is important to understanding inter-individual variability in gut microbiota. In a previous study, mouse strain-specific responses to American and ketogenic diets were observed along with several QTL for metabolic traits. In the current study, we searched for genetic variants underlying differences in the gut microbiota in response to American and ketogenic diets, which are high in fat and vary in carbohydrate composition, between C57BL/6J (B6) and FVB/NJ (FVB) mouse strains.

**Results:**

Genetic mapping of microbial features revealed 18 loci under the QTL model (i.e., marginal effects that are not specific to diet or sex), 12 loci under the QTL by diet model, and 1 locus under the QTL by sex model. Multiple metabolic and microbial features map to the distal part of Chr 1 and Chr 16 along with eigenvectors extracted from principal coordinate analysis of measures of β-diversity. *Bilophila, Ruminiclostridium 9*, and *Rikenella* (Chr 1) were identified as sex and diet independent QTL candidate keystone organisms and *Rikenelleceae RC9 Gut Group* (Chr 16) was identified as a diet-specific, candidate keystone organism in confirmatory factor analyses of traits mapping to these regions. For many microbial features, irrespective of which QTL model was used, diet or the interaction between diet and a genotype were the strongest predictors of the abundance of each microbial trait. Sex, while important to the analyses, was not as strong of a predictor for microbial abundances.

**Conclusions:**

These results demonstrate that sex, diet, and genetic background have different magnitudes of effects on inter-individual differences in gut microbiota. Therefore, Precision Nutrition through the integration of genetic variation, microbiota, and sex affecting microbiota variation will be important to predict response to diets varying in carbohydrate composition.

## Introduction

The gut microbiota has emerged as a key component underlying the application of precision nutrition and individualized dietary response. Gut microbiota utilizes nutrients passing through the gastrointestinal tract to perform biological functions, this in turn impacts host digestion, absorption, and metabolism of nutrients^1^. There is a consensus that a relationship exists between the microbes and their host. Although some studies have been performed in humans and livestock species, e.g. quails and hens^2,3^, the impact of inter-individual variability on how diet modulates gut microbiota composition remains underinvestigated^4,5^.

Previous studies from our group have demonstrated strong mouse strain-specific differences in response to American and ketogenic diets^6–9^, especially between the C57BL/6J (B6) and FVB/NJ (FVB) strains. The composition of gut microbiota is known to be influenced by both host genetics and environmental factors such as diet^9–11^, which is considered one of the most potent regulators of gut microbial composition. We have recently demonstrated that B6 is particularly susceptible to altered gut microbiota relative to A/J, FVB, and NOD/ShiLtJ^9^. Furthermore, changes to bacterial abundance do not occur uniformly in response to diets varied in macro- and micronutrient composition because of differences in substrate utilization between bacterial taxa^9,12^. To determine what the composition of the “ideal” microbiome is, it would be pertinent to disentangle the effects of host genetics and host diet from the extra layers of complexity arising from differences in substrate utilization by individual organisms and ultimately identify genes regulating interindividual differences in the composition of the gut microbiota^12,13^. Until recently, few studies have considered the extent to which the combination of host genetics and diet modulate the abundance of specific bacterial taxa and even fewer have considered how sex might add an additional layer of complexity to describing inter-individual variation in microbiota composition^14–18^.

In this study, an intercross population (F2) was generated between B6 and FVB to investigate the strain-, sex-, and diet-dependent modulation of the gut microbiota. F2s were fed either an American or a ketogenic diet and fecal microbiota was quantified. The results provide evidence for 32 quantitative trait loci (QTL) that affect microbiota composition, but also significant diet and sex differences in the effect size of the QTL. In many cases these were sex and diet independent QTL (ie, marginal effect QTL that are not specific to diet or sex, y ~ sex + diet + sex:diet + [marker]) and in other cases these were genotype- and diet-dependent (y ~ sex + diet + marker + [marker:diet]) or genotype- and sex-dependent QTL (y ~ sex + diet + marker + [marker:sex]), which allowed us to characterize how much host genetics, sex, and diet affect specific gut microbiota, and provided insights into factors driving microbial diversity, which has implications for advancing precision nutrition through preclinical studies.

## Methods

### Animals and Diets

B6 females were crossed with FVB males to generate F1 mice and subsequently intercrossed to generate an F2 population. F2s were screened for their response to American (35% of energy from fat, 50% from carbohydrates) and ketogenic (84% of energy from fat, 0% from carbohydrates) diets during a 3-month feeding trial. Detailed diet compositions are provided in Supplementary Table S1.

For the feeding trials, 3–5 week old mice were randomly assigned to one of the two diet groups and allowed to eat *ad libitum*. Half of the F2 mice were placed on American diet (102 males, 122 females) and half on ketogenic diet (126 males, 119 females). All protocols in this study were approved by the Texas A&M University Institution Animal Care and Use Committee (IACUC protocol number: 2022 – 0273). Animals were maintained at 22°C under a 12-hour light cycle with up to 5 mice per cage. At the end of the feeding trial, mice were euthanized by carbon dioxide asphyxiation, blood was collected, and tissues and feces were harvested and immediately flash frozen in liquid nitrogen.

### Microbiota Phenotypes

Stool microbiota was analyzed by 16S rRNA V4 sequencing methodology as reported previously^19^. In brief, total stool DNA was extracted using ZymoBIOMICS^™^ 96 MagBead DNA kit (Zymo Research, Irvine, CA) with an automated epMotion (Eppendorf, Hamburg, Germany) robotic system. About 100 mg of stool samples were placed in the ZR BashingBead^™^ Lysis Tube and homogenized using FastPerp24 bead beater (Millipore, Hayward, CA) at 6.5 HZ for 2 min. The lysate was centrifuged at ≥ 10,000×g for 1min and 200μl supernatant from lysis tube was transferred to 96 deep-well plate (Eppendorf, Hamburg, Germany) and loaded in an epMotion 5075t robotic system. Using epMotion, 600μl ZymoBIOMICS^™^ MagBinding Buffer and 25μl of ZymoBIOMICS^™^ MagBinding Beads were added to each well and was mixed well. After mixing, the plate was placed on a magnetic stand and the supernatant was discarded. MagBinding Beads were washed with MagWash 1 and MagWash 2 and the DNA was eluted using 50 μl ZymoBIOMICS^™^ DNase/RNase free water. The DNA concentration was measured using NanoDrop One (Thermo Scientific, Petaluma, CA).

Mixed template amplicon library for the 16S variable region 4 (V4) was prepared according to the protocol from the Earth Microbiome Project (http://www.earthmicrobiome.org/emp-standard-protocols/) using the extracted stool total DNA and the primer sets (515F and barcoded 806R)^20^. The PCR master mix, primer, and samples were plated using the epMotion. Appropriate NTC, extraction control, and pooled fecal sample were added to each plate. The PCR master mix was prepared consisting of 37.5μl of GoTaq Green Master Mix (Promega, Madison, WI), 3μl of 25mM MgCl_2_, 1.5μl of 10μM forward primer 515F, and 25.5 μL of nuclease-free water. Then, 1.5μl of 10μM barcode specific reverse primer 806R and 6μl of extracted stool DNA were added. PCR was performed in duplicate of 25μL under the following conditions: denaturation (1 cycle) at 94°C for 3 min; amplification of 25 cycles at 94°C for 45 s, 50°C for 60s, and 72°C for 90s; and a final extension step cycle at 72°C for 10min. Amplicon DNA was multiplexed and purified using Wizard SV Gel and PCR Clean-Up System (Promega, Madison, WI). The amplicon library was sequenced using the Illumina MiSEQ platform with 2×250bp paired-end sequencing. Sequences were de-multiplexed and exact amplicon sequence variants (ASV) in the 16S rRNA gene sequence were determined using the open-source software QIIME2-DADA2 pipeline^21^. A total of 11,316,115 sequences with an average of 26,074 ± 13,697 (mean ± SD) sequences per sample were recovered after demultiplexing. Taxonomy was assigned using the SILVA 132 reference database^22^ customized for 16s V4 (515F/806R) region of sequences at the threshold of 99% pairwise identity. ASV belonging to mitochondria and chloroplast were filtered out from the ASV table. We performed a single rarefaction at a sequence depth of 4,500 sequences per sample. α-diversity (Shannon diversity index and observed species) and β-diversity (unweighted UniFrac, weighted UniFrac, Jaccard Index and Bray Curtis) were calculated from the unfiltered ASV table. Any ASV not seen more than 5 times in at least 5% of the samples were removed for calculating differential bacteria abundance. 16S V4 Sequences are publicly available on the SRA database under the Bioproject ID “PRJNA803237”.

### Metabolic Phenotypes

The data analysis and collection methods for fat mass gain and serum HDL cholesterol concentration have been described previously^23^. Briefly, Echo magnetic resonance spectroscopy (MRI) (EchoMRI, Houston, TX, USA) was used to measure the fat and lean mass of all individuals. Using serum obtained from blood collected at the end of the feeding trial, total cholesterol, HDL, and LDL measurements were performed in duplicate using the EnzyChrom AF HDL and LDL/VLDL Assay kit (BioAssay Systems, Hayward, CA, USA).

### Genotyping

The genotyping analysis and collection methods have been described previously^23^. Briefly, the F2 population was genotyped on the Mouse Universal Genotyping Array (MUGA) that includes 7,854 SNP markers^24^. Markers that were not polymorphic between B6 and FVB were removed from the dataset and uncertain genotype calls for individuals (GenCall score quality metric < 0.7) were set to missing. The remaining markers were used to generate a genetic map to check for problematic markers and/or sample DNAs. After all corrections, 1,667 markers were used for the association analyses. Updated MUGA marker annotation was obtained from Dr. Karl Broman (https://kbroman.org/MUGAarrays/new_annotations.html).

### Statistical Analyses

#### Linkage Analysis

For microbiota phenotypes, a core measurable microbiota (CMM) was defined as those traits present in at least 20% of the individuals. The CMM consists of 134 ASVs. After determining organisms present in the CMM, absolute microbial abundances (counts) were quantile normalized for linkage analyses. Normal quantiles were calculated with the preprocessCore R package from Dr. Ben Bolstad, version 1.46.0 (https://github.com/bmbolstad/preprocessCore).

QTL mapping was performed on metabolic and microbial features (y) in all F2 mice from both sexes and diets, and targeted three types of effects: 1) QTL effects, whereby the effect of a marker SNP is tested after controlling for sex, diet and sex by diet interaction, which we describe in the formula as y ~ sex + diet + sex:diet + [marker], where the term in brackets is the alternative but not the null model; 2) QTL by diet effects, using y ~ sex + diet + marker + [diet:marker]; and 3); QTL by sex effects, using y ~ sex + diet + marker + [sex:marker]. QTL peaks with a logarithm of the odds (LOD) greater than thresholds determined by 10,000 permutations were considered genome-wide significant (p < 0.05, LOD > 4.00 microbial abundance, 3.90 measures of diversity) or highly significant (p < 0.01, LOD > 5.19 microbial abundance, 4.68 measures of diversity) for all models. The thresholds applied to microbial abundance and measures of diversity reflect the average genome-wide significant thresholds for all ASV present within the CMM and for all measures of diversity analyzed respectively. A LOD drop of 1.5 LOD from the top marker was used to determine the 95% confidence intervals for each QTL. Linear models using ANOVA were used to check for any interactions between sex and/or diet with the top markers of each QTL. The variance explained by the top markers at each QTL in the combined model was calculated by dividing the sum of squares of the model including the top marker by the total sum of squares of the model without QTL. The variance explained by the top markers at each QTL in the interactive models was calculated by dividing the sum of squares of the model including the interaction between diet and the top marker or sex and the top marker by the total sum of squares of the model without QTL.

Several limitations exist in the available literature for microbiome QTL analysis including the current work. Microbial data is zero-inflated compositional data^25^ and to-date no appropriate statistical method has been developed to transform and perform QTL analysis that fully addresses zero-inflation and compositional nature of the data. Zero-inflation might be the result of true biological variation or technical variation in current technologies for measuring abundances of organisms^26,27^. Of note, normalizing quantiles does not force the data into a normal distribution but rather, makes the individual microbial features more similar in statistical properties. Classical approaches to data transformation, for example the log transformation on individual ASV, similarly fail to achieve a normal distribution and otherwise substantially alter the distribution of only the non-zero data (zero data cannot be transformed). We chose to use a combination of permutation and preprocessCore’s quantile normalization across all ASV present in the CMM. Forcing the skewed data into the same distribution means that the data will behave similarly under permutation which allowed us to determine how unusual specific LOD scores were in the permutated data to identify appropriate thresholds of significance. We acknowledge a great deal of variation in methods used to normalize microbial features and even in the definition of the CMM as well as the great need to standardize these methods between investigators. Continued growth of statistical methods for zero-inflated compositional microbial data sets are needed.

### Confirmatory Factor Analysis and Structural Equation Modeling

Confirmatory factor analysis (CFA) was conducted with the lavaan R package for structural equation modeling (SEM) from Dr. Yves Rosseel, version 0.6.9 (https://www.jstatsoft.org/index.php/jss/article/view/v048i02/2448)^28^. Initial models were selected based on information from individual QTL models and correlations among traits within and between QTL models. All traits were collapsed into four, ordinal quantiles for CFA and diagonally weighted least squares estimator was used based on methods described elsewhere^29^. The final structural models illustrate QTL models for overlapping microbial and metabolic traits and are refined to include only predictors for which pathway coefficients are significantly different from zero, indicating that each of the remaining predictors in the model is significantly associating with one or more endogenous or exogenous variable.

### Candidate Gene Annotation

All genes within each significant QTL confidence interval were annotated with KEGG pathway identifiers. Candidate genes were furthered characterized by KEGG pathways related to glucose, insulin, fatty acids, adipocytes, cholesterol, obesity, diabetes mellitus, metabolic syndrome, digestion and absorption of carbohydrates, fats, and proteins, the epithelial barrier, and the immune system. A comprehensive list of KEGG pathway queries is provided in Supplementary Table S2. Transcript variants between the parental mouse strains were identified in genes annotated for selected KEGG pathways of interest from the Mouse Genome Informatics Strains, SNPS, and Polymorphisms database. Tissue specific expression was determined with Mouse ENCODE Transcriptome data, accessed through the National Library of Medicine National Center for Biotechnology database.

## Results

### Diet is a strong modulator of the gut microbiome

**Figure 1:**
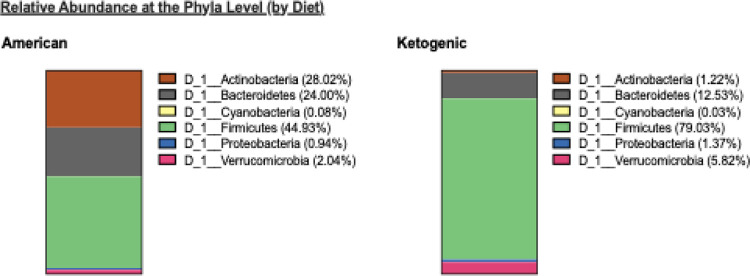
Relative abundance of microbiota and correlations at the phyla level (by diet). **A**. Relative abundance of Firmicutes in F2s on the ketogenic diet is nearly twice as high as the relative abundance of Firmicutes in F2s on the American diet at the expense of Actinobacteria, and Bacteroidetes for which the relative abundances are lower in F2s on the ketogenic diet.

Diet explains a large proportion of variation in the abundance of microbiota at the Phyla level irrespective to genetic background. Diet explains 64.79% of variation in abundance of Actinobacteria, 61.22% of variation in the abundance of Firmicutes, and 25.49% of variation in the abundance of Bacteroidetes (Supplementary Table S3). The relative abundance of Firmicutes in F2 on the ketogenic diet is nearly twice as high as in F2 on the American diet (Fig. 1). This increase in Firmicutes in F2 on the ketogenic diet appears to occur at the expense of the relative abundance of Actinobacteria and Bacteroidetes (Fig. 1).

**Figure 2:**
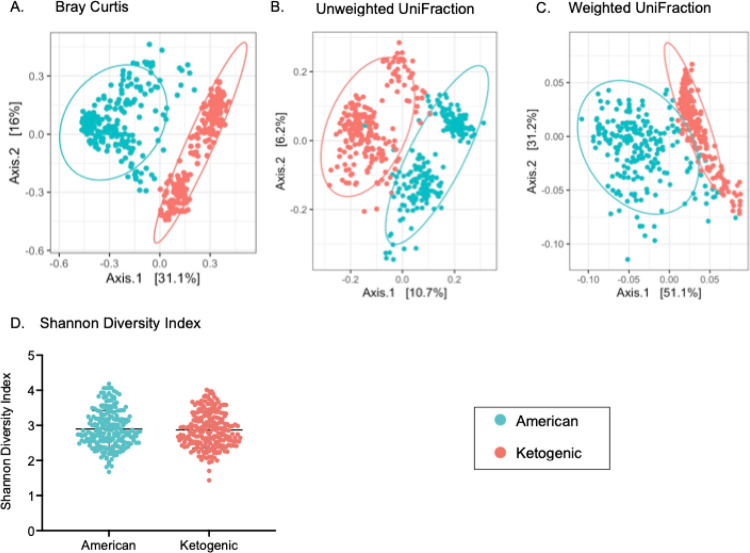
Beta Diversity Principal Coordinate Analysis (PCoA). **A. Bray Curtis Index**. PC1 and PC2 describe 31.1% and 16% of the variation in ASV respectively. **B. Unweighted UniFraction**. PC1 and PC2 describe 10.7% and 6.2% of the variation in ASV respectively. **C. Weighted UniFraction**. PC1 and PC2 describe 51.1% and 31.2% of the variation in ASV respectively. Overlaying diet with the meausres of beta-diversity illustrates two distinct groups, roughly segregating PC1 for all measures of beta diversity. **D. Shannon Diversity Index**. The Shannon Diversity Index is not different between diet groups.

Principal coordinate analysis (PCoA) for measures of beta diversity revealed two distinct groups segregating at PC1. The Bray-Curtis index PCo1 and PCo2 describe 31.1% and 16% of the variation in ASV respectively (Fig. 2A). The Jaccard Index PCo1 and PCo2 are nearly identical to the Bray-Curtis index and describe 21.3% and 10.6% of variation in ASV respectively (Supplementary Figure S2). Unweighted UniFraction PCo1 and PCo2 describe 10.7% and 6.2% of variation in ASV respectively (Fig. 2B). Weighted UniFraction PCo1 and PCo2 describe 51.1% and 31.2% of variation in ASV respectively (Fig. 2C). Overlaying diet with the data illustrates two distinct groups roughly segregating PCo1 for all measures of beta diversity. However, alpha diversity, illustrated by the Shannon Diversity Index, does not depend on diet (Fig. 2D). Eigenvectors extracted from the PCo1 and PCo2 from the Bray-Curtis index, Jaccard index, unweighted and weighted UniFractions, as well as values from the Shannon Diversity Index served as additional traits for linkage analysis below.

### Microbial features are modulated by genetic loci

**Figure 3:**
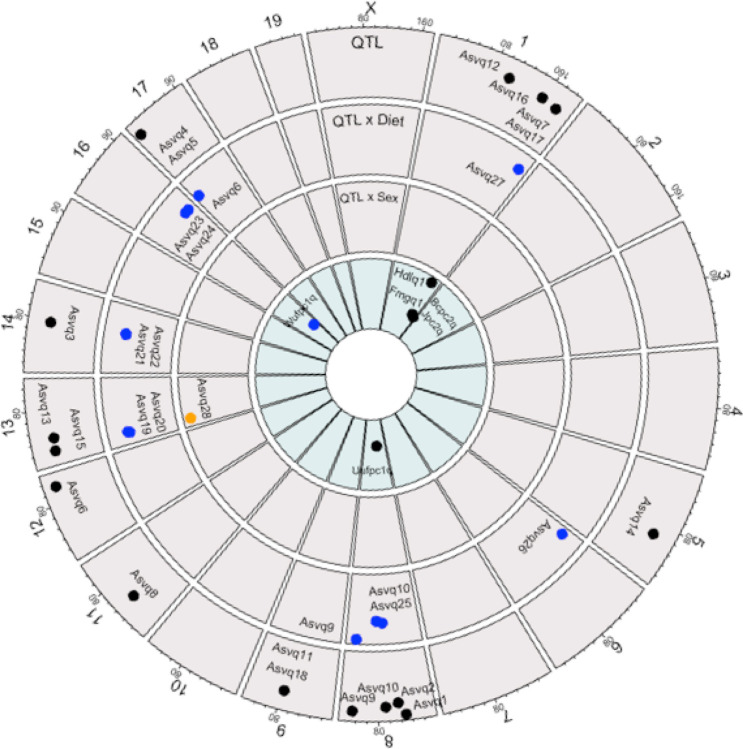
Microbial QTL. Outer ring to inner ring: Significant QTL in the QTL model (black) for ASV associated with the genus *Coriobacteriaceae UCG-002* on Chr 8 at *Asvq1*, the genus *Enterohabdus* on Chr 8 at *Asvq2*, multiple uncultured bacterium from the *Muribaculaceae* family on Chr 14 at *Asvq3* and Chr 17 at *Asvq4* and *Asvq5*, the genus *Alistipes* on Chr 12 at *Asvq6*, the genus *Rikenella* on Chr 1 at *Asvq7*, the genus *Rikenellaceae RC9 Gut Group* on Chr 11 at *Asvq8*, the genus *Streptococcuson* Chr 8 at *Asvq9* and *Asvq10*, the genus *GCA-900066575* on Chr 9 at *Asvq11*, the genus *Lachnoclostridium* on Chr 1 at *Asvq12* and Chr 13 at *Asvq13*, the genus *Romboutsia* on Chr 5 at *Asvq14 and* Chr 13 at *Asvq15*, the genus *Ruminiclostridium 9* on Chr 1 at *Asvq16*, and the genus *Bilophila* on Chr 1 at *Asvq17* and Chr 9 at *Asvq18*. Significant QTL in the diet specific model (blue) for ASV associated with multiple uncultured bacterium from the *Muribaculaceae* family on Chr 13 at *Asvq19* and *Asvq20*, Chr 14 at *Asvq21 and Asvq22*, and Chr 17 at *Asvq5*, the genus *Rikenelleceae RC9 Gut Group* on Chr 16 at *Asvq23*, the genus *Parabacteroides* on Chr 16 at *Asvq24*, the genus *Lactobacillus* on Chr 8 at *Asvq25*, multiple ASV from the *Streptococcus* genus on Chr 8 at *Asvq9*, and *Asvq10*, the uncultured genera from the family of *Clostridiales vadinBB60 group* on Chr 6 at *Asvq26*, and the *Lachnospiraceae* family on Chr 1 at *Asvq27*. A single significant QTL in the sex specific model (orange) for the genus *Alistipeson* Chr 13 at *Asvq28;* Previously identified QTL for metabolic traits and diversity measures in the combined model (black) and diet specific model (blue). Fat mass gain during the feeding trial on Chr 1 at *Fmgq1*, along with serum HDL cholesterol concentration after the feeding trial on Chr 1 at *Hdlq1*, Bray-Curtis and Jaccard measures of beta diversity at *Bcpc2q* and *Jpc2q*. Unweighted unifraction on Chr 8 at *Uufpc2q*, and weighted unifraction on Chr 16 at *Wufpc1q. Fmgq1, Hdlq1, Bcpc2q*, and *Jpc2q* overlap the same region of the genome as *Asvq7, Asvq16*, and *Asvq17* for uncultured *Rikenella, Ruminiclostridium*, and uncultured *Bilophila. Wufpc1q* overlaps the same region of the genome for *Asvq23* and *Asvq24* for *Rikenelleceae RC9 gut group* and *Parabacteroides*.

In the sex and diet-independent QTL model, which tests for marginal effect QTL after controlling for sex and diet (see Methods; y ~ sex + diet + sex:diet + [marker]), 18 distinct QTL were detected for 15 unique microbial abundances (counts), leaving 119 additional organisms within the CMM not displaying a genetic linkage (Fig. 3, Table 1).

*Asvq7*, for the genus *Rikenella* overlaps with *Asvq16* and *Asvq17* for *Ruminiclostridium 9* and *Bilophila* genera, respectively, as well as with the previously identified QTL for fat mass gain (*Fmgq1*) and serum HDL cholesterol concentration (*Hdlq1*).

Apart from *Coriobacteriaceae* (*Asvq1*), diet appears to explain a more significant proportion of the variation in the abundance of these ASV despite this QTL not being diet specific. This is the only ASV with a QTL for which the top marker explains a greater proportion of the variation in the abundance of the organism than diet itself despite there being a significant effect of diet as well in this model (Table 1). For all the remaining loci identified under the QTL model, diet explains a greater proportion of the variation than the top marker does at each QTL. This is particularly clear for *Streptococcus* where diet explains nearly 50% of the variation in abundance of two *Streptococcus* ASVs while the top markers at *Asvq9* and *Asvq10* explain only 3.14% and 2.92% of the variation, respectively (Table 1).

### Microbial features are modulated by diet-specific genetic loci

The analysis for the interaction between QTL and diet detected 12 QTL for 11 unique microbial features, leaving 123 organisms in the CMM not displaying diet-specific genetic linkage (Fig. 3, Table 1). Nine of these QTL were distinct from the ones identified in the QTL model.

Of note, three diet specific QTL were identified that are identical or nearly identical to *Asvq5, Asvq9*, and *Asvq10* that were identified in the QTL model for the same organisms. There are only modest differences in the 95% confidence intervals at *Asvq9* and *Asvq10* in the loci identified in the QTL and QTL by diet models. The top marker is unchanged between the QTL and QTL by diet models at all 3 loci so these loci will continue to be referred to as *Asvq5*, *Asvq9*, *Asvq10*.

Interestingly, diet alone explains a greater proportion of the variation than the interaction between diet and the top marker at the diet specific QTL for *Muribaculeceae* (*Asvq19, Asvq20, Asvq21, Asvq22, Asvq5*), *Rikenellaceae RC9* Gut Group (*Asvq23*), *Streptococcus* (*Asvq9, Asvq10*), and the uncultured geneus of the *Lachnospiraceae* family (*Asvq27*) (Table 1). The interaction between genotype and diet appears to explain the greatest proportion of variation at *Asvq24* for *Parabacteroides* (4.11%), *Asvq25* for *Lactobacillus* (4.22%), and *Asvq26* for *Clostridiales* VadinBB60 Group (4.56%), (Table 1).

### Microbial features are modulated by sex-specific genetic loci

The QTL by sex analysis detected a single significant QTL for *Alistipes* on Chr 13 at 18.4 Mb (*Asvq28*) (Fig. 3, Table 1).

Sex explains 1.66% of variation in the abundance of *Alistipes* while the interaction between sex and the top marker at *Asvq28* explains over 4% of variation in the abundance of this OTU (Table 1). Even in the sex-specific model of *Asvq28*, diet explains a greater proportion of the variation than either sex or the interaction between sex and the genotype.

### Measures of Beta diversity are genetically modulated

In the QTL model, three distinct QTL were identified for PCo2 of several measures of beta-diversity including Bray-Curtis, Jaccard, and the unweighted UniFraction (Fig. 3, Table 2). The eigenvectors for both Bray-Curtis and the Jaccard index PCo2 map to Chr 1 at 177.5 Mb (Bcpc2q; p < 0.05, CI = 160.6–185.1 Mb; Jpc2q; p < 0.05, CI = 160.6 = 185.1 Mb), overlapping with Asvq7 (151.9–193.3 Mb), Asvq16 (138–186.3 Mb), Asvq17 (144.5–193.3 Mb) and previously identified QTL for fat mass gain (Fmgq1; 180–194.4 Mb) and serum HDL cholesterol concentration (Hdlq1; 160.6–176.1 Mb)^23^. As noted above, PCo2 of Bray-Curtis and the Jaccard index appears to be modestly represented by alpha diversity. The remaining locus identified by the QTL model was for unweighted UniFraction PC2 at 53.7 Mb (Uwufpc2q; p < 0.05, CI = 43.4–62.8 Mb). No QTL were identified for the Shannon Diversity Index.

In the QTL by diet analysis, one additional QTL was identified for weighted UniFraction PC1 on Chr 16 at 79.6 Mb (Wufpc1q; p < 0.01, 95% CI = 72.8–95.8 Mb), overlapping with Asvq23 and Asvq24 for the Rikenelleceae RC9 Gut Group and Parabacteroides (Asvq23; 72.8–96.5 Mb, Asvq24; 72–96.5 Mb).

### Candidate keystone species modulating the microbiome and physiological traits

**Figure 4:**
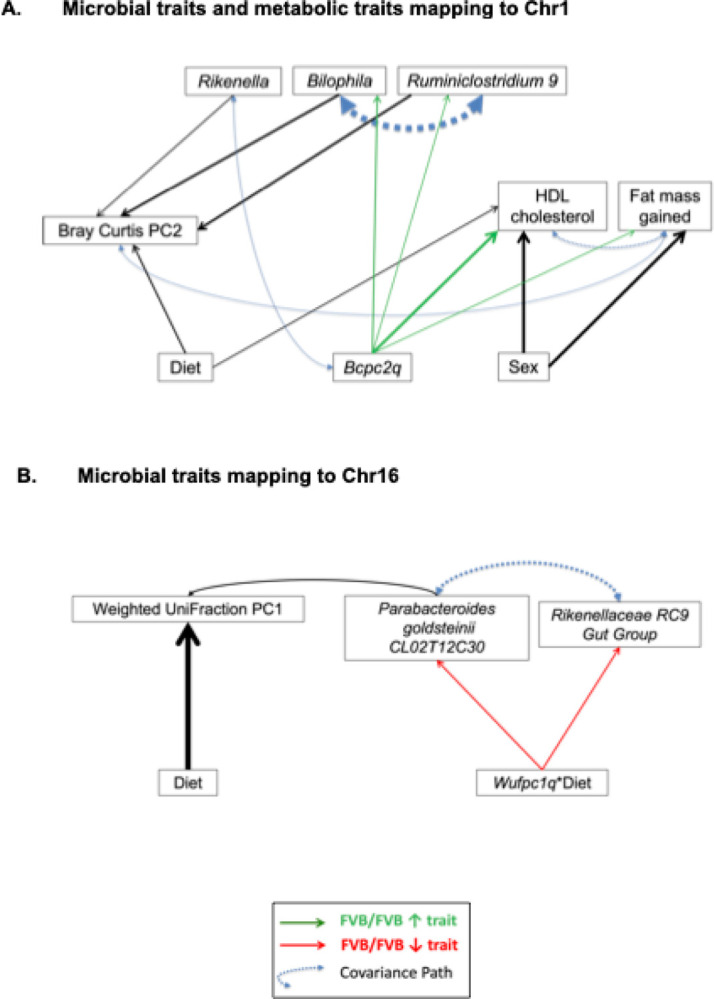
Graphical Representation of SEM. Solid, single headed arrows indicate the direction of paths and the weight of each arrow is proportional to the path coefficient (r) from the predictor to the variable and the percentage of variation in the variable that is explained by each predictor. Positive effects (green arrows) indicate that the FVB allele increases the trait; negative effects (red arrows) indicate that the FVB allele decreases the trait. Double headed, blue arrows represent covariate pathways detected in the structural model of microbial features and measures of diversity.

A structural equation model (SEM) was built to illustrate the magnitude of the effects of each predictor in the models of *Bilophila, Rikenella, Ruminiclostridium 9*, and Bray-Curtis PCo2, all mapping to the distal part of Chr 1 (Fig. 4A). Genotypes at *Bcpc2q* were chosen to model all traits mapping to the distal part of Chr 1 as *Bcpc2q* is contained inside of the confidence interval for the other three loci mapping to the distal region of Chr 1. The initial model was refined until path coefficients were all significantly different from zero, suggesting that each of the remaining predictors in the model is significantly associating with one or more of the other predictors. (Table 3). The refined model suggests that the FVB/FVB genotype at *Bcpc2q* increases abundances of *Bilophila, Ruminiclostridium 9*, and *Rikenella*, and these three ASV are driving differences in the Bray-Curtis index PCo2 (Fig. 4A). A covariance pathway is detected among abundances of *Bilophila* and *Ruminiclostridium 9* in addition to their individual, direct effects on the Bray-Curtis index. The inclusion of metabolic traits does not elucidate direct, indirect, or covariance pathways between the metabolic traits and specific organisms mapping to distal Chr 1. However, a covariance pathway is observed between the Bray-Curtis index PCo2 and the amount of fat mass gained.

Similarly, a SEM was built to illustrate the magnitude of effects of each predictor in the models of *Parabacteroides, Rikenellaceae RC9 gut group*, and weighted UniFraction PCo1, all mapping to distal Chr 16 (Fig. 4B). Genotypes at *Wufpc1q* were chosen for the model as the 95% confidence interval for this locus is contained within the QTL of all other traits in this structural model. The path coefficients are again, all significantly different from zero (Table 3). The refined model suggests that the interaction between the FVB/FVB genotype and the American diet at *Wufpc1q* directly decrease abundances of *Parabacteroides* and the *Rikenelleceae RC9 Gut Group*. The abundance of *Parabacteroides* has a direct relationship with the weighted UniFraction PCo1. A covariance pathway is detected among abundances of *Parabacteroides* and the *Rikenelleceae RC9 Gut Group*.

### Identification of candidate genes at Asvq7, Asvq16, Asvq17, and Bcpc2q

Candidate genes that might elucidate the relationship between *Rikenella, Ruminiclostridium 9, Bilophila*, and the Bray-Curtis PCo2 were investigated. *Bcpc2q* is contained inside of the confidence interval for the other three loci mapping to the distal region of Chr 1. Positional candidates at *Bcpc2q* that overlap with one or more metabolic KEGG pathways are summarized in Table 4. Out of 275 positional candidates at *Bcpc2q*, 35 genes overlap with one or more KEGG pathways. Eleven out of the total 35 positional candidates harbor known non-synonymous transcript variants diverging between these strains. The presence of these non-synonymous transcript variants makes *Aim2, Apoa2, Atp1a4, Cadm3, Cd244a, Cd48, F11r, Fcer1g, Mpz, Ndufs2, Sdhc*, and *Sell* the primary candidate genes of interest in this region. Of these, *F11r, Fcer1g, Ndufs2* and *Sdhc* are expressed in the intestine.

### Identification of candidate genes at Asvq23, Asvq24, and Wufpc1q

Candidate genes were investigated that might elucidate the relationship between *Rikenelleceae RC9* Gut Group, *Parabacteroides*, weighted UniFraction, *Asvq23, Asvq24*, and *Wufpc1q*. Positional candidates at *Wufpc1q that* overlap with one or more metabolic KEGG pathways are summarized in Table 4. Out of 133 positional candidates at *Wufpc1q* 11 genes overlap with one or more metabolic KEGG pathways. However, none of the genes annotated with KEGG pathway from our query harbor known non-synonymous transcript variants diverging between the two strains.

## Discussion

This study provides evidence that abundances of gut microbiota are driven by unique combinations of effects from the host’s genetics, response to high fat diets varied in carbohydrate content, and sex. Many previous studies have compared the effects of control mouse diets to high fat diets where one or two representative ingredients contribute to the total fat, carbohydrate, and protein content of the diet^6^. The American and ketogenic diets used here more accurately recapitulate the diversity of ingredients found in human diets not only in terms of the macronutrient profile of human diets but also the fiber content and lipid profiles^6^. The diverse set of ingredients is particularly important to studies of the microbiota because of differences in substrate utilization between bacterial taxa. There is an abundance of literature supporting the potent effects of diet on the abundance of gut microbiota driven by fiber, carbohydrate, protein, and lipid source and composition^9,11,12, 16–18,30–32^. Other studies have demonstrated that the effect of abnormal diets on gut microbiota might stifle the underlying effect of single gene mutations because diets are such a potent regulator of microbial abundances^16,33^. These authors have called for further study of diets varied in macronutrient content and study of more complex genetic models. The current study has demonstrated that high fat diets varied in carbohydrate content continue to be commanding predictors of abundances of gut microbial abundances even in a more complex genetic model. We will highlight below many results for which genetic effects are likely dependent on specific ingredients in one of the two diets and discuss the importance of incorporating human-comparable diets into microbial studies.

Another unique feature of the current study is the incorporation of latent variables harbored in the PCoA of ASV data. Latent variables are those that are not directly observable in a model but can be inferred from other variables and can hold important information for interpreting biological relationships. PCoA of ASV data revealed that PCo1 captures the variance in ASV caused by diet while it was less clear what variance was captured by PCo2. Extraction of eigenvectors from these principal components is one way to incorporate information from the latent variables contained in the PCoA.

In this study, 18 loci diet and sex-independent loci were identified. Out of these loci, Coriobacteriaceae is the least influenced by diet. Coriobacteriaceae is the only ASV for which the top marker explains a greater proportion of the variation in the abundance of the organism than diet, suggesting that there is a genetic predisposition to having higher or lower abundances of Coriobacteriaceae. Coriobacteriaceae has previously been associated with host genetics and QTL regulating immune function and susceptibility to carcinoma and tumor development in mice^34,35^. Coriobacteriaceae has been described as a dominant species in the mammalian gut and it is positively correlated with hepatic triglyceride concentration and non-HDL cholesterol concentration in mice^36^.

A significant proportion of the variation in all other ASV with loci detected by the diet and sex-independent model is still driven by diet, especially for Streptococcus at Asvq9 and Asvq10. Streptococcus belongs to the Firmicutes phylum. Fiber is a particularly important dietary component for modulating abundance of Firmicutes. When animals switch from a low fat/fiber rich plant diet to a high fat/high sugar diet, they experience a significant increase in the Firmicutes phylum along with a decrease in Bacteroidetes^17^. Dramatic shifts were observed in these phyla between American and ketogenic diet F2s irrespective of their genetic backgrounds. Our ketogenic diet is composed of twice as much soluble and insoluble fiber as the American diet, and this likely drives many of the differences in the abundance of OTUs from these phyla. The relative abundance of Firmicutes in F2s exposed to the ketogenic diet is nearly twice as high as F2s exposed to the ketogenic diet. It appears that this increase in Firmicutes in the F2s exposed to the ketogenic diet coincides with a decrease in the relative abundance of Bacteroidetes. Limited evidence suggests that a higher Firmicutes to Bacteroidetes ratio is positively correlated with obesity while a decrease in this ratio has been associated with inflammatory bowel disease^37^. However, controversy surrounds the association of the Firmicutes to Bacteroidetes ratio and health status^37^.

Six diet-specific QTL were also identified under the QTL by diet model. Of note, all six diet-specific QTL are for microbial features from either the Firmicutes or Bacteroidetes phyla. These QTL include, Asvq19, Asvq20, Asvq21, Asvq22, and Asvq5 for Muribaculaceae (Bacteroidetes), Asvq23 for Rikenelleceae RC9 Gut Group (Bacteroidetes), Asvq24 for Parabacteroides (Bacteroidetes), Asvq25 for Lactobacillus (Firmicutes), Asvq9 and Asvq10 for the Streptococcus genus (Firmicutes), Asvq26 for the family of Clostridiales vadinBB60 (Firmicutes), and Asvq27 for the Lachnospiraceae family (Firmicutes). This provides further support for previous findings suggesting that the ratio of Firmicutes to Bacteroidetes is relevant to metabolic disease states. Diet is the strongest predictor for these ASV except for the abundances of Parabacteroides, Lactobacillus, and Clostridiales vadin BB60 (all Firmicutes). The gene-by-diet interaction is most prominent for these three exceptions. Diet-specific QTL are the most clinically actionable observations as they identify a subgroup of the population that would be sensitive to a dietary intervention to modify the microbial trait. Loci identified by the sex and diet- independent QTL and QTL by sex models are informative but do not provide the same type of direct avenue for intervention.

The American diet contains multiple sources of animal proteins, some of which contribute to the total fat content of the diet, while the main protein source in the ketogenic diet is casein. The fat component of the ketogenic diet is composed of equal parts butter and lard with a small portion of corn and menhaden oils, while the fat component of the American diet is a more diverse mixture of primarily butter as well as corn, menhaden, flaxseed, olive oil, and fat derived from the animal proteins. Lard-derived fat has been shown to reduce the abundance of Streptococcus^11^. This may explain, in part, why we observe that FVB alleles on the American diet are associated with higher abundances of two Streptococci ASV.

For the only loci picked up by the QTL by sex model, Asvq28 for Alistipes, the strongest predictor in the model was again diet. Sex specificity for abundance of Alistipes has been established in studies of pre- and-post-menopausal women and men. Men were more likely than pre- or post-menopausal women to have higher abundances of Alistipes in their fecal samples^38^. The realized importance of sex as a biological variable has increased attention paid to the role of steroid hormones in development of obesity and Metabolic Syndrome^23^. Plasma testosterone has also been linked to microbial features in men, and the post-menopausal microbiome becomes more similar to the male microbiome over time^38^.

QTL for Rikenella (Asvq7), Ruminiclostridium (Asvq16), Bilophila (Asvq17), Bray-Curtis PCo2 (Bcpc2q), fat mass gained during the feeding trial (Fmgq1), and serum HDL cholesterol concentration (Hdlq1) overlap on the distal part of Chr 1 and QTL for the Rikenelleceae RC9 Gut Group (Asvq23), Parabacteroides (Asvq24) and weighted UniFraction PCo1 (Wufpc1q) overlap on Chr 16. American or westernized diets are associated with increased abundances of Bilophila wadsworthia, which coincides with increased LDL cholesterol concentration and links this species of Bilophila to dyslipidemia and increased inflammation^39^. Gut microbiota signatures from overweight and obese patients have been associated with significant decreases in Rikenella and Parabacteroides species as well as increases in Ruminococcus species in the same subjects^40^. Rikenellaceae RC9 gut group has been associated with lipid metabolism in response to high fat diets^15,41^. Previous associations between these organisms and metabolic traits make the overlapping loci associated with them higher priority for future analyses.

As mentioned previously, gut microbiota utilizes nutrients passing through the gastrointestinal tract. Microbial metabolism of these nutrients produces metabolites and microbial-derived metabolites known to impact metabolic health^1^. These metabolites may represent latent variables linking the genomic region underlying Fmgq1, Hdlq1, Bcpc2q, Asvq7, Asvq16, and Asvq17 and each of their associated traits as well as Wufpc1q, Asvq23, and Asvq24 and their associated traits.

The SEM for traits mapping to distal Chr 1 illustrated direct effects of the FVB/FVB genotype at Bcpc2q increasing abundance of Bilophila and Ruminiclostridium 9. A covariance pathway was detected between Bcpc2q and Rikenella and while the directionality of this relationship was not defined by the model, this suggests that the FVB/FVB genotype at Bcpc2q also increased the abundance of Rikenella. We observed direct effects of Bilophila, Ruminiclostridium 9 and Rikenella on Bray Curtis PC2 in addition to the direct effect of diet. In addition to the direct effects of Bilophila and Ruminiclostridium 9, we identified a covariance pathway between these organisms that likely contributes to the overall relationship of these microbiota with Bray-Curtis PC2. While the microbiota and metabolic traits appear to be independently linked to Bcpc2q, another covariance pathway is observed between Bray-Curtis PCo2 and the amount of fat mass gained during the feeding trial. Taken together, these observations suggest that Bilophila, Ruminiclostridium 9, and Rikenella are driving differences in microbial beta-diversity represented in Bray-Curtis PCo2, and the overall composition of the microbiome may be correlated with the amount of fat mass gained during the feeding trial. Species that other species in an ecosystem rely heavily upon are referred to as keystone species and drive diversity within the ecosystem^42,43^. These results suggest that Bilophila, Ruminiclostridium 9, and Rikenella are candidate keystone species. The covariance pathway observed between the Bray-Curtis index PCo2 and the amount of fat mass gained might reflect a more complex relationship between the overall composition of the gut microbiota and its effects on metabolic features.

The SEM for traits mapping to distal Chr 16 illustrated direct effects of Wufpc1q interacting with diet on the abundances of Rikenellaceae RC9 Gut Group and Parabacteroides as well as a direct effect of diet on weighted UniFraction PCo1. We observed a covariance pathway between these two organisms as well as between Rikenellaceae RC9 Gut Group and weighted uniFraction PCo1. This suggests Rikenellaceae RC9 Gut Group may be an additional candidate keystone species driving differences in the composition of the microbiota in a diet-specific manner.

Fluctuations in the abundance of these organisms would have dramatic consequences on other organisms in the ecosystem. Bilophila, Ruminicostridium 9 and Rikenella represent candidates for keystone species among the organisms mapping to the distal region of Chr 1. Their direct effects on Bray-Curtis PCo2 detected in the structural equation model suggest abundances of these organisms drive differences in beta diversity. The proposed model lends itself to this speculation if the abundance of Bilophila has consequences for bile acid composition and abundances of other microbiota in the large intestine as described by others^44,45^. We have previously demonstrated that the FVB/FVB genotype drives higher serum HDL cholesterol concentration at the locus Hdlq1, likely through Apolipoprotein A2 (Apoa2)^23^. Apoa2 is also a primary candidate gene of interest within the confidence intervals for Asvq7, Asvq16, Asvq17, and Bcpc2q. HDL cholesterol is a preferred precursor to bile acid synthesis and secretion^46^. Despite there being no direct relationship observed between abundance of these organisms and metabolic traits in the current model, these basic biological associations leave ample space for future analyses into what is likely a more complex network of latent variables tying together these microbial and metabolic traits. Additional candidate genes of interest that are expressed in the intestines were identified within the Bcpc2q interval (F11r, Fcer1g, Ndufs2 and Sdhc). F11r and Fcer1g are found on KEGG pathways primarily related to the immune system while Ndufs2 and Sdhc are found on the Non-alcoholic fatty liver disease pathway (mmu04932).

Parabacteroides was also identified as a candidate keystone species among the organisms mapping to the distal part of Chr 16. We were unable to narrow the list of positional candidate genes at Wufpc1q harboring non-synonymous transcript variants with the KEGG pathways included in the query. However, the vast majority of genes at Wufpc1q were annotated with the epithelial barrier and immune system pathways such as Tight junction (mmu04530) and Inflammatory bowel disease (mmu05321) and related pathways. Other types of variants were present in genes within the Wufpc1q confidence interval such as, synonymous transcript variants and intronic variants which may be of interest in future analyses. For example, Sod1 harbors an intron variant that diverges between the two strains and has previously been associated with both the ratio of Firmicutes to Bacteroidetes as well as obesity, providing direct evidence for variants in Sod1 regulating microbial diversity and a possible link to metabolic traits like obesity^47,48^. Of note, Parabacteroides and Rikenelleceae RC9 gut group both belong to the Bacteroidetes phylum. Future work will focus on confirming causal relationships between candidate keystone species and measures of beta-diversity.

## Conclusions

The current experiment identified organisms for which irrespective to genetic background, diet was the strongest predictor of gut microbiota, organisms for which combinations of sex, diet, and genotypes predictor the gut microbiota, as well as a single organism for which genetic background was the strongest predictor for bacterial, Coriobacteriaceae UCG-002. These results demonstrate the effect that sex, diet, and genetic background have on inter-individual differences in gut microbiota. While diet and genotype-dependent QTLs for microbial abundance are the most clinically relevant regarding efforts to advance precision nutrition, diet-dependent observations are likely related to specific ingredients in the diets which makes these observations heavily context dependent and difficult to recapitulate from investigator to investigator when non-human comparable ingredients are used in the preclinical setting. We observed that nearly all microbial QTL, even those that were identified under the QTL and QTL by sex models, were potently influenced by diet. As such, care should be taken to utilize diets composed of diverse ingredients in preclinical trials to better recapitulate the host-microbiome environment in humans. Precision nutrition will be advanced through integration of genetic variation, microbiota variation, and sex in response to diets varied in carbohydrate composition to elucidate the composition of the “ideal” microbiome and personalized interventions to achieve that composition.

## Data Availability

16S V4 Sequences are publicly available on the SRA database under the Bioproject ID “PRJNA803237”.
